# Bisphenol A-Induced Epigenetic Changes and Its Effects on the Male Reproductive System

**DOI:** 10.3389/fendo.2020.00453

**Published:** 2020-07-30

**Authors:** Federica Cariati, Luigi Carbone, Alessandro Conforti, Francesca Bagnulo, Stefania Ramona Peluso, Consolata Carotenuto, Cira Buonfantino, Erminia Alviggi, Carlo Alviggi, Ida Strina

**Affiliations:** ^1^CEINGE-Biotecnologie Avanzate s.c.a.r.l., Naples, Italy; ^2^Fertility Unit, Maternal-Child Department, AOU Policlinico Federico II, Naples, Italy; ^3^Department of Neuroscience, Reproductive Sciences and Odontostomatology, Federico II University, Naples, Italy; ^4^Molecular Medicine and Medical Biotechnology Department, Federico II University, Naples, Italy; ^5^Department of Public Health, Federico II University, Naples, Italy; ^6^GENERA Centers for Reproductive Medicine, Clinica Ruesch, Naples, Italy; ^7^Endocrinology and Experimental Oncology Institute (IEOS), National Research Council, Naples, Italy

**Keywords:** bisphenol A, male reproduction, infertility, epigenetic, oxidative stress, DNA methylation, spermatogenesis, prostate cancer

## Abstract

Bisphenol A (BPA) is a widespread chemical agent which can exert detrimental effects on the male reproductive system. Exposure to BPA has been shown to induce several epigenetic modifications in both animal and human cells. Specifically, BPA could not only modify the methylation pattern of multiple genes encoding proteins related to reproductive physiology but also directly influence the genes responsible for DNA methylation. BPA effects include hormonal alterations, microscopic and macroscopic alteration of male reproductive organs, and inheritable epigenetic changes involving human reproduction. BPA exposure was also linked to prostate cancer. This review aims to show the current scenario of BPA-induced epigenetic changes and its effects on the male reproductive system. Possible strategies to counter the toxic effect of BPA were also addressed.

## Introduction

Epigenetics is the science that studies the environmental influence over the genetic heritage without modifying the DNA sequence. Epigenetic modifications include chromatin remodeling, histone modifications, and non-coding RNA mechanisms, which in turn could affect the phenotype of different types of cells. Epigenetics acts in the regulation of the expression of silencing genes in response to a variety of environmental exposures, allowing cells to answer and adapt to environmental stressors (1). Moreover, epigenetic modifications in parents can determine long-lasting changes, evolving in pathologies for the offspring (1).

It is now acknowledged that widespread chemical agents can exert detrimental effects on human physiology. Some substances identified as “endocrine-disrupting chemicals” (EDC) could also interfere with the endocrine system (2–4).

Bisphenol A (BPA) is an organic synthetic compound largely used for the production of polycarbonate plastics and epoxy resins. Due to the widespread industrial use of polycarbonate plastics (e.g., food/drink packaging, production of compact discs, impact-resistant safety equipment, and medical devices), the presence of BPA is ubiquitous (5). BPA can be detected in various body fluids like urine, saliva, blood, breast milk, and amniotic fluid, as well as on the skin (6). The most harmful effect of BPA is due to both its estrogenic and anti-estrogenic properties (7, 8). BPA is able to bind to multiple targets both inside and outside the nucleus, inducing alterations in various endocrine-related pathways (7). The effects of BPA on the hypothalamic–pituitary–gonadal axis, determining pathologic consequences on the reproductive system, have been elucidated in animal and human studies (9). In addition to short-term effects, it has been demonstrated that BPA can alter epigenetic mechanisms producing also long-term effects (1).

The ability of BPA to alter normal epigenetic patterns has been recently demonstrated. Some studies revealed a role in the differentiation of spermatogenic cells, through the functional modification of some genes (10, 11). This review intends to summarize the epigenetic mechanisms by which BPA acts on both animal and human male reproductive systems. Furthermore, the possible strategies to counteract BPA effects were also disclosed and discussed.

## Materials and Methods

The search was conducted using Medline, Embase, Web of Science, Scopus, ClinicalTrials.gov, Ovid, and Cochrane Library as electronic databases. Studies were identified using the combination of the following search terms: “bisphenol A” AND “epigenetic” OR “epigenetic changes” OR “male” OR “male reproduction” OR “reproduction” OR “male reproductive system” OR “sperm” OR “sperm function” OR “sperm changes” OR “spermatogenesis” OR “prostate cancer” OR “oxidative stress” OR “offspring” OR “transgenerational” OR “transgenerational effects” OR “transgenerational changes” OR “DNA methylation,” from inception of each database to May 2020. Therefore, all data from both animal and human studies on the relationship among BPA and the different aspects of the male reproductive system were considered for inclusion. However, only data regarding epigenetic changes were included in the review. All discrepancies were resolved by discussion among authors. No restrictions for language were applied. Unpublished studies were not included. Data were presented and categorized in relation to the different level at which BPA may induce impairment of male reproductive system physiology. The list of the genes mentioned throughout the whole text is presented with their proper nomenclature and role in Table 1.

**Table 1:** Genes studied in relation to BPA exposure and male reproductive system alterations.

**Acronym**	**Gene**	**Role**	**Reference paragraph**
DNMT3A	DNA methyltransferase 3 alpha	*De novo* methylation	Reproductive endocrine system Spermatogenesis impairment Risk of prostatic cancer
DNMT3B	DNA methyltransferase 3 beta	*De novo* methylation	Reproductive endocrine system Spermatogenesis impairment Risk of prostatic cancer
ERα	Estrogen receptor α	Sexual development and reproductive function	Reproductive endocrine system
H19	Imprinted maternally expressed transcript 19	• Imprinted gene only expressed from the maternally inherited chromosome • Epigenetic changes in this gene have been associated with Beckwith–Wiedemann syndrome • Epigenetic deregulations at H19 imprinted gene in sperm have been observed associated with male infertility	Reproductive endocrine system Spermatogenesis impairment Transgenerational effects
IGF2	Insulin-like growth factor 2	• Imprinted gene only expressed from the paternally inherited chromosome • Epigenetic changes at this locus are associated with Wilms tumor, Beckwith–Wiedemann syndrome, rhabdomyosarcoma, and Silver–Russell syndrom	Reproductive endocrine system Spermatogenesis impairment Transgenerational effects
StAR	Steroidogenic acute regulation protein	Regulation of steroid hormone synthesis by enhancing the conversion of cholesterol into pregnenolone	Reproductive endocrine system
P450scc	Cytochrome P450 family	Drug metabolism and synthesis of cholesterol, steroids, and other lipids	Reproductive endocrine system
CYP17A1	Cytochrome P450 family	Drug metabolism and synthesis of cholesterol, steroids, and other lipids	Reproductive endocrine system
3β-HSD	3β-Hydroxysteroid dehydrogenase	Catalyzation of the oxidative conversion of delta (5)-ene-3-beta-hydroxy steroids and the oxidative conversion of ketosteroids	Reproductive endocrine system
EXPO5	Exportin 5	Transport of small RNAs and double-stranded RNA-binding proteins from the nucleus to the cytoplasm	Teratogenesis and gonadal morphology
DICER	Ribonuclease type III	Production of small RNA component that represses gene expression	Teratogenesis and gonadal morphology
DROSHA	Ribonuclease type III	MicroRNA (miRNA) synthesis	Teratogenesis and gonadal morphology
AGO2	Argonaute RISC catalytic component 2	Short-interfering-RNA-mediated gene silencing	Teratogenesis and gonadal morphology
DNMT3L	DNA methyltransferase 3 like	• *De novo* methylation • Transcriptional repression	Spermatogenesis impairment
H3K9	Histone H3-lysine 9	• Involvement in acetylation of genes for activation, methylation of genes for silencing • Marker of heterochromatin	Spermatogenesis impairment Transgenerational effects
H3K4	Histone H3-lysine 4	Involvement in acetylation of genes for activation, methylation of genes for silencing	Spermatogenesis impairment
DNMT1	DNA methyltransferase 1	*De novo* methylation	Spermatogenesis impairment Risk of prostatic cancer
H3K9Me3	Histone H3-lysine 9	• Trimethylation at the 9th lysine residue of the histone H3 protein • Binding heterochromatin protein 1 (HP1) to constitute heterochromatin	Spermatogenesis impairment
H3K27Me3	Histone H3-lysine 27	• Trimethylation at the 27th lysine residue of the histone H3 protein • Involvement in the peroxisome-associated pathway and induction of peroxisome loss to ameliorate oxidative stress	Spermatogenesis impairment
H3K9Me1	Histone H3-lysine 9	• Monomethylation at the 9th lysine residue of the histone H3 protein	Spermatogenesis impairment
H3K9Me2	Histone H3-lysine 27	• Dimethylation at the 9th lysine residue of the histone H3 protein • Mark of the inactivated X chromosome (Xi)	Spermatogenesis impairment
MYBPH	Histone H3-lysine 9	Biased expression in prostate	Spermatogenesis impairment
PRKCD	Protein kinase C δ	Tumor suppressor and cell cycle progression	Spermatogenesis impairment
IGF2R	Insulin-like growth factor 2 receptor	• Intracellular trafficking of lysosomal enzymes • Activation of transforming growth factor beta • Degradation of insulin-like growth factor 2	Spermatogenesis impairment
G9a	Lysine methyltransferase	Key histone methyltransferase for H3K9me1 and H3K9me2	Spermatogenesis impairment
GNMT	Glycine N-methyltransferase	Catalyzation of the conversion of S-adenosyl-L-methionine (along with glycine) to S-adenosyl-L-homocysteine and sarcosine	Spermatogenesis impairment
TET	Ten–eleven translocation protein	Regulation of DNA demethylation, gene transcription, embryonic development, and oncogenesis	Spermatogenesis impairment
LINE-1	Long interspersed nucleotide elements 1	• Gene regulation by the 5′ UTR methylation level • Active in germ cells and silent in most of the somatic cells	Spermatogenesis impairment
ACHE	Acetylcholinesterase	Hydrolyzation of the neurotransmitter acetylcholine in choline and acetic acid	Spermatogenesis impairment
H3K27	Histone H3-lysine 27	• Epigenetic mark • Regulation of chromatin structure and gene expression	Transgenerational effects
H4K12	Histone H4-lysine 12	• Epigenetic mark • Regulation of chromatin structure and gene expression	Transgenerational effects
SIRT1	Sirtuin 1	Regulation of epigenetic gene silencing and suppression of rDNA recombination	Transgenerational effects
ERβ	Estrogen receptor β	• Transcription activation • Inhibition of the activity of other estrogen receptor family members	Transgenerational effects
CAV-1	Caveolin 1	Involvement in the Ras-ERK pathway and promotion of cell cycle progression	Transgenerational effects
IGF2R	Insulin-like growth factor 2 receptor	• Intracellular trafficking of lysosomal enzymes • Activation of transforming growth factor beta • Degradation of insulin-like growth factor 2	Transgenerational effects
PEG3	Paternally expressed 3 gene	• Paternally expressed • Involvement in cell proliferation and p53-mediated apoptosis	Transgenerational effects
SLC12A2	Na-K-Cl cotransporter	Mediation of sodium and chloride transport and reabsorption	Risk of prostatic cancer
PDE4D4	Phosphodiesterase 4D4	3′,5′-Cyclic-AMP phosphodiesterase activity and cAMP degradation	Risk of prostatic cancer
HPCAL1	Hippocalcin-like 1	Calcium-dependent regulation of rhodopsin phosphorylation with implication in neuronal signaling in the central nervous system	Risk of prostatic cancer
MBD2	Methyl-CpG-binding domain protein 2	• Binding specifically to methylated DNA sequences • Transcription repression from methylated gene promoters • Mediation of the biological consequences of the methylation signal	Risk of prostatic cancer
GPCR14	Putative G-protein coupled receptor	Mediation of signaling processes to the interior of the cell via activation of heterotrimeric G proteins	Risk of prostatic cancer
PDGFRα	Platelet-derived growth factor receptor alpha	Mitogenesis for cells of mesenchymal origin	Risk of prostatic cancer
PLCβ3	Phospholipase C beta 3	Catalyzation of the diacylglycerol and inositol 1,4,5-triphosphate from phosphatidylinositol in G-protein-linked receptor-mediated signal transduction	Risk of prostatic cancer
NSBP1	Nucleosomal binding protein 1	Nucleosomal binding and transcriptional activating protein	Risk of prostatic cancer
HMGN5	High-mobility group nucleosome-binding domain 5	Nucleosomal binding and transcriptional activating protein	Risk of prostatic cancer
PITX3	Paired-like homeodomain 3	Lens formation during eye development	Risk of prostatic cancer
WNT10B	Wnt family member 10B	• Oncogenesis • Regulation of cell fate and patterning during embryogenesis	Risk of prostatic cancer
PAQR4	Progestin and adipoQ receptor family member 4	Tumor suppression by inhibition of the Raf/MEK/ERK signaling cascade	Risk of prostatic cancer
SOX2	SRY-box transcription factor 2	Regulation of embryonic development and determination of cell viability	Risk of prostatic cancer
CHST14	Carbohydrate sulfotransferase 14	Catalyzation of sulfate transfer to the C-4 hydroxyl of N-acetylgalactosamine residues in dermatan sulfate	Risk of prostatic cancer
TPD52	Tumor protein D52	Tumor progression	Risk of prostatic cancer
CREB3L4	CAMP-responsive element-binding protein 3 like 4	Adiposity and male germ cell development	Risk of prostatic cancer
EZH2	Enhancer of zeste 2 polycomb repressive complex 2 subunit	Maintaining of the transcriptional repressive state of genes over following cellular generations	Risk of prostatic cancer
UHRF1	Ubiquitin-like with PHD and ring finger domains 1	Regulation of gene expression	Risk of prostatic cancer
BCR	Breakpoint cancer region	• Serine/threonine kinase activity • GTPase activation of protein for p21rac and other kinases	
PTGS2	Prostaglandin-endoperoxide synthase 2	Involvement in prostaglandin biosynthesis	Risk of prostatic cancer
TIMP3	Tissue inhibitor of metalloproteinase 3	Inhibition of the matrix metalloproteinases with a role in tumor suppression	Risk of prostatic cancer
ZMYDN10	Loss of zinc finger MYND-type containing 10	Tumor suppressor	Risk of prostatic cancer
GSTP1	Glutathione S-transferase Pi 1	Detoxification by catalyzing the conjugation of many hydrophobic and electrophilic compounds with reduced glutathione	Risk of prostatic cancer
LOX	Lysyl oxidase	Tumor suppression	Risk of prostatic cancer
MGMT	O-6-Methylguanine-DNA methyltransferase	Cellular defense against mutagenesis and toxicity from alkylating agents	Risk of prostatic cancer
NEUROG	Neurogenin 1	Transcriptional regulator	Risk of prostatic cancer
TSC2	TSC complex subunit 2	Tumor suppression	Risk of prostatic cancer
PDLIM4	PDZ and LIM domain 4	Bone development	Risk of prostatic cancer
PYCARD	PYD and CARD domain containing	Mediation of signaling complex assembly in the inflammatory and apoptotic signaling pathways via the activation of caspase	Risk of prostatic cancer
KDM5B	Lysine demethylase 5B	Transcriptional repression	Risk of prostatic cancer
NSD1	Nuclear receptor-binding SET domain protein 1	Androgen receptor transactivation	Risk of prostatic cancer

### Reproductive Endocrine System

BPA is able to bind hormonal receptors, stimulating, or inhibiting the physiologic pathway. Consequently, the ability to interfere with the hormonal axis has been observed, thereby influencing steroid signaling (12). BPA affects testis competence, varying the gene expression of steroid hormone receptors and influencing the enzymes that catalyze DNA methylation, as demonstrated by *in vivo* and *in vitro* animal studies (13–21).

In fishes (adult males of rare minnow *Gobiocypris rarus*), BPA has been demonstrated to affect the gene expression of steroid hormone biosynthesis, blood–testis barrier, proteolysis, lipid transport, and metabolism (13).

In rats, similar data were obtained, showing how BPA exposure influences the hypothalamic–pituitary–gonadal axis, finally modifying the levels of steroid hormone receptors in testes, with important consequences on sperm parameters as motility and count (14). Again, when neonatal male rats were exposed to BPA for the first 5 days of life, a change in gene expression of estrogen receptors (ERα and ERβ) in adult testis and an increase in both transcript and protein levels of DNA methyltransferases (DNMT3A and DNMT3B) were revealed (15). Interestingly, El Henafy et al. (16) obtained very similar results, analyzing the methylation pattern of DNMT3A and ERα, showing hypermethylation for both genes, in male rat pups exposed to BPA by transplacental and trans-lactational routes. In addition, their findings indicated that if the period of exposure was longer (pregnancy plus lactation), the effects were higher, suggesting a dose–response effect (16).

The interference of BPA with hormones is also suggested from an *in vivo* study in mice, where a flavonoid-based diet was administered to counteract the epigenetic effects induced by BPA. DNA methyltransferase expression was inhibited, with a decrease in epigenetic methylation of ERα and H19/IGF2 genes (the H19 imprinting is associated with IGF2 since they have the same gene locus and common enhancers) and of reproductive hormone levels, thus contrasting BPA's effect (17).

Another mechanism by which epigenetic changes are induced may be the increase in oxidative stress caused by BPA exposure (18, 19); in this sense, an *in vitro* study from Zhang et al. (20) showed that after exposing mouse testicular cells to BPA, the mRNA levels of proteins involved in sexual hormone steroidogenesis as StAR, P450scc, Cyp17a1, and 3β-HSD were reduced but were also normalized after exposure to melatonin. Finally, the exposure to BPA in mouse preimplantation embryo produces a disruption of testicular synthesis of testosterone and reduction of StAR promoter histone acetylation, thereby inducing a retard of testis development (21).

### Teratogenesis and Gonadal Morphology

The majority of the studies principally focus on molecular mechanisms of pathophysiological changes and not on proper structural abnormalities. However, some evidence from animal studies showed how the BPA exposure promotes teratogenesis and affects testis morphology.

In zebrafish embryo–larvae, BPA shows teratogenic properties, provoking different anomalies going from cardiac edema to craniofacial abnormalities, spinal malformations, cranial hemorrhage, and yolk sac deformity, depending on dose of exposure (22).

In mice, BPA administration appears to compromise the testis morphology; especially the size of seminiferous tubules and the epithelium were significantly reduced with impairment of spermatogenesis at various stages (21).

Moreover, El Henafy et al. (16) evidenced that BPA could significantly impair anogenital distance, which represents an important measure of genital development, as well as testis and epididymis weight.

Another study showed the involvement of Sertoli cells, essential for physical and nutritional support of developing germ cells, as a target of epigenetic and transcriptome alterations from environmental toxicant exposures. These epigenetic alterations are related to testis abnormalities (23).

Cho et al. studied the influence of BPA on micro-RNA (miRNA): in mouse Sertoli cell lines, the BPA was shown to alter miRNA expression, with subsequent gene expression modification, and related changes in reproductive patterns (24).

An *in vitro* study on testicular fragments culture from 7-day-old male pigs exposed to BPA demonstrated a downregulation of EXPO5 and Dicer genes and an upregulation of Drosha and AGO2 genes, involved in miRNA pathways. Also, Leydig cells' morphology was not altered but interstitial tissue collagen was increased (25).

### Spermatogenesis Impairment

Epigenetic modifications can occur at different steps during spermatogenesis. Firstly, primordial germ cells are subjected to genomic imprinting through a process of DNA/histone demethylation and deacetylation of H4 (Histone 4). DNA methyltransferases already expressed at this stage are DNMT3A, DNMT3B, and DNMT3L. Then, *de novo* DNA methylation occurs in spermatogonia and remains stable until fertilization and zygote development (26). This mechanism appears necessary to complete spermatic meiosis, as suggested in a study in which DNA methyltransferase knockout mice resulted to be sterile because they were unable to sustain meiosis (26). Furthermore, H3K9 and H3K4 methylation takes place in spermatocytes; DNMT1 is expressed in round spermatids, where hyperacetylated H4 is found and replacement of histone variants by transition proteins is starting. Elongated spermatids show establishment of a DNA methylation pattern associated with histone H3K9 demethylation. The histone-to-protamine transition is completed at this stage of spermatogenesis. Finally, the genomic imprinting is saved in spermatozoa (27). The histone-to-protamine transition permits a packaging of spermatic DNA, with condensation of sperm heads and protection of DNA from damage and mutagenesis. However, a low percentage of histones can remain in sperm, at undefined genes or gene promoter levels, causing possible post-translational modifications, resulting in severely altered reproductive phenotypes (11, 28, 29). It has been demonstrated that the alteration of the histone–protamine ratio affects male fertility (27).

In mice, *in vitro* studies on testis germ cells exposed to high doses of BPA demonstrated a decrease in the global DNA methylation levels, due to a reduction in DNMT1 protein and mRNA. At the same time, histone hypomethylation of H3K9Me3, H3K27Me, H3K9Me1, and H3K9Me2 was revealed. These changes seem to be mediated by a reduction in G9a proteins, which are essential methyltransferases for the meiotic process and hence for the whole spermatogenesis (20, 30).

The toxic effect of BPA on mouse semen quality was demonstrated from Zhang et al., who observed an increased number of morphologically altered and headless spermatozoa; in addition, sperm motility was reduced, after subcutaneous injection or feeding with BPA (31). Yin et al. demonstrated the alteration of DNA methylation of MYBPH and PRKCD, eliciting a change in spermatocyte proliferation and motility in a murine model (32).

In fishes, several studies showed that BPA exposure causes an impairment of global DNA methylation in the testes and consequently reduced rate of fertilization (33–36).

In details, in *Gobiocypris rarus*, BPA-induced DNA hypermethylation was demonstrated and explained by several mechanisms, including *de novo* synthesis of glutathione and oxidative stress, in addition to a significant decrease of the TET protein levels, responsible for demethylation (33, 36, 37). It was also observed that administration of antioxidants as N-acetylcysteine may reverse such damages, protecting DNA integrity and sperm motility (36).

On the contrary, in zebrafish gonads, a global DNA demethylation due to a transcriptional miss-regulation of the DNA methylation/demethylation-associated genes (DNMTs, GNMT, and TETs) was noticed (35, 38). Moreover, a compromised spermatogenesis in male zebrafish exposed to a high dose of BPA was demonstrated. As a matter of fact, a significant decrease in sperm count was seen together with an increase in apoptosis; in addition, a miss-regulation of transcription of enzymes responsible for epigenetic remodeling was proven, leading to an increase in histone acetyltransferase activity and causing alterations in embryo development (34, 39).

In *Danio rerio* zebrafish, Lombó et al. observed sperm DNA fragmentation dependent on dose and time of BPA exposure (6).

In humans, the dimethylation of histone H3 on lysine K4 has been demonstrated to be negatively correlated with sperm concentration, motility, and mitochondrial function (40). In particular, a genome-wide study on semen samples from workers exposed to BPA and unexposed controls showed the ability of that compound to interfere with gene expression during spermatogenesis, with DNA hydroxymethylation due to H3 trimethylation, clinically ending in reduced sperm concentration motility (41). More recent data confirm previous findings, especially demonstrating a LINE-1 hydroxymethylation (42). Since LINE-1 activation has already been studied in relation to male infertility, its epigenetic modifications induced by BPA exposure may be one of the mechanisms for this EDC's toxicity. In another study, blood and semen samples collected from BPA exposed vs. non-exposed men were analyzed, in order to evaluate the toxic effect on a marker of genome-wide methylation status as LINE-1. Results showed a significantly lower methylation level of sperm LINE-1 in workers exposed to BPA. In addition, the BPA urinary levels were associated with low semen quality, even though they were inversely correlated with LINE-1 methylation (43).

Men exposed to BPA showed an increase in the rate of 5-hydroxymethylcytosine (5-hmc, which is a marker of DNA demethylation processes and demonstrates active gene transcription) of the sperm ACHE gene. Therefore, the accumulation of 5-hmc is associated with demethylation status. Taking into consideration that this type of alteration is correlated with sperm concentration and motility, the authors suggested that male infertility could be a consequence of BPA exposure (10, 44). Indeed, the effects of BPA on spermatogenesis are widely discussed in literature, whereas the majority of the studies do not explicitly mention if the underlying pathogenetic mechanisms are epigenetic (5, 14).

Moreover, since the effects of BPA exposure are also unfolded by DNA damage and epigenetic modifications, information on the influence of BPA on spermatogenesis and related male infertility is derived not only by studies directly analyzing sperm parameters but also through evidence of embryo and offspring abnormalities, as for transgenerational effects, thus described accordingly.

### Transgenerational Effects

The process of DNA methylation is closely linked to the well-known phenomenon of genomic imprinting, wherein a gene is differentially expressed depending on whether it has been inherited from the mother or from the father. Examples of imprinting-derived diseases are Angelman syndrome and Prader-Willi syndrome. These, although caused by the epigenetic modification of the same gene, elicit different consequences depending on which parent it has been inherited from (45).

When a “safe” dose of bisphenol A was administered for a long time in rats, a decrease in histone acetylation of H3K9, H3K27, and H4K12, an increase in deacetylase Sirt1 expression with reduced binding, and finally an increase in estrogen receptor β (ERβ) to caveolin-1 (Cav-1) binding were observed. These processes and the related findings provided clues about the underlying mechanisms for epigenetic inheritance induced by BPA exposure (46).

An indirect proof of alteration of the sperm epigenome came from the study of Doshi et al., who evaluated the percentage of post-implantation loss and expression of DNMTs in embryos of pregnant female rats coupled with BPA-exposed males. They pointed out that post-implantation loss rate appeared to be higher and resorbed embryos had lower expression of DNMTs when sired by BPA-exposed males, compared to viable embryos from both BPA-exposed and control males (47). In addition, in their following work on the imprinting control region (ICR) of two genes implicated in embryonic growth and cellular proliferation, H19 and IGF2, the methylation pattern was analyzed. The authors showed hypomethylation at the H19-ICR in both spermatozoa and resorbed embryos from neonatally BPA-exposed rats, demonstrating that epigenetic mechanisms regulate both infertility, and transmission to offspring (48).

Oppositely, Zhang et al. noticed no changes in methylation of IGF2, IGF2R, Peg3, and H19, which are imprinted genes. However, they acknowledged that the offspring of BPA-exposed mice had smaller size and worse pelage quality, thus admitting a certain effect of this compound (31).

Shi et al. demonstrated how BPA modifies the mRNA expression of DNA and histone methyltransferases and their associated factors in the testis of a generation of mouse neonates prenatally exposed to that compound and how these effects were transmitted to the third generation of offspring (49).

In *Danio rerio* zebrafish, treatment with BPA during embryogenesis did not impact the methylation profile of sperm, although a decrease in H3K9ac, involved in sperm development, was observed (50).

An *in vivo* study on adult zebrafish males exposed to BPA during spermatogenesis and mated with non-exposed females revealed a disruption of cardiogenesis in forthcoming generations (51).

Akhter et al. studied the appearance of different malformations in various generations of zebrafish, after that the parental generation was exposed to BPA, finding abnormalities in the testes of the second-generation males and explaining this as a trans-generational effect most probably due to epigenetic mechanisms (52).

Other lines of evidence from animal studies showed that sperm motility was associated with methylation variation affecting genes involved in chromatin organization. The result of this alteration could affect embryo development (53, 54).

In a study on perinatal exposure of pregnant rats to BPA, the authors observed male fertility impairments in the three subsequent generations (13).

Hong et al. observed a reduction in the population of all sperm cells at different stages of development (spermatogonia, spermatocytes, and spermatids) in adult mouse testes, after exposure of preimplantation embryos to low-dose BPA, suggesting it as a consequence of epigenetic mechanisms (21).

Moreover, male rats subjected to neonatal BPA exposure showed downregulation of DNMT gene expression and related transcription factors, with impact on sperm epigenome and therefore influence on embryo development and implantation process (47).

In addition, after fetal exposure to BPA *in utero*, male rats were mated with unexposed female rats: the results showed an epigenetic alteration of IGF2 methylation in the male germline and subsequently promotion of glucose intolerance and β-cell dysfunction in the offspring, proving therefore the inheritance of epigenetic pattern changes, leading to dysregulation and disease (55, 56).

Furthermore, a study on pregnant rats exposed to environmental compounds including BPA during embryonic gonadal sex determination showed pubertal abnormalities, testis disease, obesity, and ovarian disease in the third generation. Apoptosis of spermatogenic cells resulted to be impaired through different generations of offspring (57). Moreover, 197 differential DNA methylation regions (DMR) in the gene promoter were shown in the sperm epigenome in the third generation after exposure. Authors stated that the sperm DMRs could represent epigenetic biomarkers for transgenerational disease and/or ancestral environmental exposures (58).

### Risk of Prostatic Cancer

Ho et al. identified 28 genes as possible markers of epigenetic modifications, looking in particular to DNA methylation, leading to increased predisposition to adult-onset prostate cancer in rats, after neonatal estrogenic or BPA exposure. The majority of such genes were implicated in signal transduction pathways: Na–K–Cl cotransporter (SLC12A2), mitogen-activated protein kinase (MAPK)/extracellular signal-regulated kinase (ERK) pathway (GPCR14 and PDGFRα), phosphokinase C pathway (PLCβ3), and cAMP pathways (PDE4D4 and HPCAL1). In particular, the prostatic PDE4D4 gene remains expressed in all rats early exposed to a low dose of BPA, before adult-onset prostatic lesions; in addition, HPCAL1 showed a specific methylation and expression alteration with aging. Therefore, the authors concluded that early exposure to BPA could provoke permanent impairment of the prostate epigenome, determining a predisposition to prostate cancer (59–62). Later, Tang et al. showed that few genes, such as DNMT3A, DNMT3B, and MBD2, responsible for epigenetic mechanisms, were overexpressed in rats after early exposure to BPA. Moreover, this study defined three patterns of epigenetic changes, characterizing genes like NSBP1 and HMGN5, persistently present epigenetic markers of early-life exposure; a second group represented by PDE4D4, which appear only at genital maturation but persist throughout life; and the last group, including genes such as HPCAL1 considered modifiable epigenetic markers, whose later appearance depends on early-exposure features and subsequent events during adult life (63). Moreover, Cheong et al. analyzed the prostatic tissue of BPA early-exposed rats for the methylation pattern of 7 genes (PITX3, WNT10B, PAQR4, SOX2, CHST14, TPD52, and CREB3L4), at the promoter region, showing that 4 of them (PITX3, WNT10B, PAQR4, and TPD52) were differently methylated when comparing prostatic cancer cells with normal adjacent tissues. They also noticed a connection with recurrence-free survival of prostatic cancer patients (64). Interestingly, Prins et al., discovered that different prostatic regions and lobes in rats have variable sensitivities to different doses of early-administered BPA in later-developing cancerous lesions, with different dose-dependent methylation patterns: CREB3L4, TPD52, and PITX3 showed a noteworthy hypomethylation at lower doses of BPA, with a normalization toward higher doses; PAQR4 showed significant hypomethylation for all BPA doses; and SOX2 showed an inverse correlation between hypomethylation and BPA doses (65). In a study on healthy primary human prostate epithelial cells (PrECs) exposed to high concentrations of BPA and analyzed using a whole-genome microarray, the authors noticed that BPA can modify the expression of epigenetic factors as EZH2, DNMT1, DNMT3B, and UHRF1, producing transcriptional perturbations with epigenetic consequences and even raising cancer risks (66). In addition, Karaman et al., studying prostatic carcinoma cells, observed hypermethylation in the p16 promoter region as well as for BCR, PTGS2, TIMP3, and ZMYDN10, with different changes seen in GSTP1, LOX, MGMT, NEUROG, and TSC2 methylation pattern. Also, a low dose of BPA could determine hypomethylation of PDLIM4 and PYCARD. Moreover, exposure to BPA induces downregulation of chromatin-modifying enzymes like KDM5B and NSD1 (67).

## Discussion

Our review intended to highlight the mechanisms by which BPA modifies at various levels the reproductive system. In particular, we looked into literature and summarized the studies that analyzed the epigenetic changes leading to impairment of the different aspects of male reproduction, both in animals and in humans.

Epigenetics is responsible for the control of many genes implicated in hormonal production, sperm parameters, and inheritable abnormalities (Tables 2–6).

**Table 2:** Characteristics of the studies which analyzed hormonal axis disturbances.

**Epigenetic modifications**	**Effects**	**Study type**	**Species**	**References**
Genes expression	• Induction of gene expression in the renin–angiotensin system pathway • Inhibition of tRNA processing-related gene expression • Decreases in hemostasis and blood coagulation-related gene expression	*In vivo*	Fish	(13)
DNA methylation	• Hypermethylation of ERα/ERβ promoter regions • Increase in DNMT3A and DNMT3B expression	*In vivo*	Rat	(15)
DNA methylation	Hypermethylation within DNMT3A and ER∞	*In vivo*	Rat	(16)
DNA methylation	• Hypermethylation of the ERα promoter and H19/Igf2 imprinting control region in the testis • Increase of DNMT expression	*In vivo*	Mouse	(17)
DNA methylation	• Decrease in G9a-dependent H3K9 di-methylation • Impairment of spermatogenesis	*In vitro*	Mouse	(20)
Histone acetylation	• Decrease in H3 and H3K14 acetylation in the StAR and P450 in the testes • Decrease in the expressions of testicular StAR and P450scc	*In vivo*	Mouse	(21)

**Table 3:** Characteristics of the studies which analyzed morphological alterations.

**Epigenetic modifications**	**Effects**	**Study type**	**Species**	**References**
Histone acetylation	Reduction in diameter and epithelium height of seminiferous tubules and spermatogenic cells at different stages	*In vivo*	Mouse	(21)
Gene expression	Upregulation or downregulation of 37 miRNA related to overexpression of genes implicated in metabolism and reproduction	*In vitro*	Mouse	(24)
miRNA biogenesis and function	• No changes in Leydig cell morphology • No changes in lipid droplet content and distribution • Changes in lipid and autophagy protein abundance • Downregulation of EXPO5 and Dicer genes and an upregulation of Drosha and AGO2 genes	*In vitro*	Boar	(25)

**Table 4:** Characteristics of the studies which analyzed the epigenetic impairment of spermatogenesis.

**Epigenetic modifications**	**Effects**	**Study type**	**Species**	**References**
Histone methylation	• Decrease of DNMT • Reduction in the global DNA methylation levels in spermatogonia	*In vitro*	Mouse	(30)
DNA methylation	• No effect on DNA methylation of imprinted genes (IGF2, IGF2R, PEG3. and H19) in germ cells • Increase in ERα expression • Impairment of meiotic progression of germ cells • Decrease in quality and quantity of spermatozoa	*In vivo*	Mice	(31)
DNA methylation	• Reduction in DNA replication capacity • Alteration of the genome-wide DNA methylation level in GC-2 cells • Alteration of DNMT expression levels • Regulation of MYBPH and PRKCD methylation	*In vivo*	Mouse	(32)
DNA methylation	Promotion of the DNA methylation process in the testes by novo synthesis of glutathione and oxidative stress	*In vivo*	Fish	(33)
DNA methylation	Alteration of the global DNA methylation level of gonads	*In vivo*	Fish	(34)
DNA methylation	• Alteration of the global DNA methylation level of gonads • Transcriptional change of genes (DNMTS, GNMT, and TEST)	*In vivo*	Fish	(35)
DNA methylation	Variation in DNA methylation levels	*In vivo*	Fish	(36)
DNA methylation	Hypermethylation of global DNA in the testes	*In vivo*	Fish	(37)
DNA methylation	Global DNA demethylation	*In vivo*	Fish	(38)
DNA methylation	• Decrease of spermatocytes • Increase in apoptosis • Downregulation of CCNB1 and SYCP3 • Upregulation of GPER1 and ESRRGA receptors • Miss-regulation of epigenetic remodeling enzyme transcripts • DNA hypermethylation • H3K27me3 demethylation • Increase in histone acetyltransferase activity	*In vitro*	Fish	(39)
DNA methylation	• Di-methylation of lysine K4 on histones H3 • Impairment of motility, concentration, and mitochondrial activity in sperm	*In vivo*	Human	(40)
DNA methylation	Trimethylation of histone 3 (H3K27me3, H3K4me2, or H3K4me3) in sperm	*In vivo*	Human	(41)
DNA methylation	Hypomethylation of LINE-1	*In vivo*	Human	(42)
DNA methylation	• Decrease in sperm LINE-1 methylation status • Association between BPA urinary levels and low semen quality	*In vivo*	Human	(43)
DNA methylation	• Correlation between 5hmC rates of AChE and low sperm motility • Correlation between HoxC4 promoters and sperm concentration	*In vivo*	Human	(44)

**Table 5:** Characteristics of the studies which analyzed the transgenerational effects of BPA exposure.

**Epigenetic modification**	**Effects**	**Study type**	**Species**	**References**
Histone acetylation DNA methylation	Apoptosis and impairment of the meiotic process	*In vitro*	Fish	(39)
DNA methylation	• Decrease in histone acetylation of H3K9, H3K27, and H4K12 • Increase in protein expression of deacetylase Sirt1 • Reduction in binding of Sirt1 and ERβ to caveolin-1	*In vivo*	Mouse	(46)
DNA methylation	Downregulation of the gene expression of DNMTS and related transcription factors	*In vivo*	Rat	(47)
DNA methylation	• Hypomethylation of the H19 imprinting control region • Downregulation in the transcript expression of IGF2 and H19	*In vivo*	Rat	(48)
DNA methylation	• Expression of DNMT3A in Sertoli cells • Strengthening of DNMT3B and weakening H3K9me2 and H3K9me3 in germ cells of the neonatal testis	*In vivo*	Mouse	(49)
DNA methylation	• Impairment of primordial germ cell (PGC) migration to the genital ridge • Dysregulation of genes involved in PGC migration (CXCR4B and SDF1A) • No alteration of DNA methylation	*In vivo*	Fish	(50)
Gene expression	• Increase in the rate of heart failure of progeny up to the second generation deriving from females that mated with males exposed to BPA • Downregulation of 5 genes involved in cardiac development in first-generation embryos • Decrease in parents and first-generation sperm remnant mRNAs related to early development	*In vivo*	Fish	(51)
DNA methylation	Maintenance of chromosome structure through epigenetic regulation correlated with sperm functionality	*In vivo*	Bull	(53)
DNA methylation	Hypermethylation of IGF2, glucose intolerance, and β-cell dysfunction in islets in offspring	*In vivo*	Rat	(55)
DNA methylation	Global DNA methylation decreased in the first-generation sperm	*In vivo*	Rat	(56)
DNA methylation	Sperm DMR correlation with several adult-onset pathologies (e.g., mammary tumors, prostate disease, kidney disease, testis abnormalities, immune abnormalities) in offspring	*In vivo*	Rat	(57)

**Table 6:** Characteristics of the studies which analyzed the risk of prostate cancer induced by BPA exposure.

**Epigenetic modification**	**Effects**	**Study type**	**Species**	**References**
DNA methylation	Hypomethylation of the prostate cancer gene (PDE4D4)	*In vivo*/*In vitro*	Human	(59)
DNA methylation	• Aberrant NSBP1 promoter demethylation and transcriptional overexpression persisting in adult life • Aberrant HPCAL1 promoter hypermethylation and transcriptional suppression with a little degree of gene expression in adult life • High expression of DNMT3A and DNMT3B in early life, diminishing with aging • Involvement in early-life reprogramming of DNA methylation patterns in target genes such as NSBP1 or HPCAL1	*In vitro*	Rat	(63)
DNA methylation	DNA methylation-mediated gene expression of 6 genes linked to embryonic stem cell pluripotency	*In vivo*	Rat	(64)
DNA methylation	DNA hypomethylation of genes that confer carcinogenic risk	*In vivo*	Rat	(65)
DNA methylation	Deregulation of EZH2, DNMT1, DNMT3B and UHRF1	*In vitro*	Human	(66)
DNA methylation	• Expression levels of p16 gene decreased significantly after promoter hypermethylation • p16-related histone modifications • Dose-dependent promoter hypermethylation of tumor suppressor genes as BCR, PTGS2, TIMP3, and ZMYDN10 • Hypomethylation of PDLIM4 and PYCARD • Demethylation of GSTP1, LOX, MGMT, NEUROG, and TSC2 • Significant decrease of gene expression levels and downregulation of KDM5B and NSD1 measured in RT-PCR (real-time polymerase chain reaction)	*In vitro*	Human	(67)

The interesting point that comes out from our analysis is that BPA acts on two levels of epigenetic changes. In fact, on the one hand, it is responsible for the widely altered DNA methylation, the most commonly studied epigenetic mechanism; on the other hand, studies showed that the main effect of bisphenol A is on genes related to methylation proteins. In other words, BPA might be considered as an example of a proper epigenetic controller.

In this paper, we have also illustrated the possible strategies to counteract the epigenetic effect of BPA. Indeed, several antioxidants can ameliorate reproductive function by inhibiting BPA's effect on oxidative stress (68–70).

Since the increase in ROS (reactive oxygen species) is one of the recognized effects of BPA in male spermatogenesis, causing reduction in sperm viability and motility, due also to mitochondrial dysfunction, a study explored the efficacy of taurine in reversing such events, although not properly epigenetic changes, observing good results in a dose-dependent fashion (71). N-Acetylcysteine also has been evaluated to reduce ROS after BPA exposure, showing amelioration of sperm motility (36).

As abovementioned, flavonoids can defend from the epigenetic modifications induced by bisphenol A, due to their antioxidant and similar estrogenic properties (17). In addition, thanks to its antioxidant and free radical scavenger properties, melatonin has been demonstrated to pass the blood–testis barrier and protect steroidogenesis and spermatogenesis, acting principally on H3K9me2 and DNA methylation (20, 72).

Folates are methyl donors, essential for the DNA methylation process and for stabilization of the methylation status of the epigenome. Mao et al. (73) studied the efficacy of folate supplementation during pregnancy in restoring pancreatic function after BPA administration in rats, obtaining a reversal of its epigenetic changes. Moreover, Dolinoy et al. (74) demonstrated that supplementation with folate or phytoestrogen as genistein during pregnancy could counteract the effects of BPA exposure in *Agouti* mice, showing reduction in the hypomethylation pattern and hence pelage modification. These studies support that the transgenerational effects of BPA could be reduced by folate administration.

In 2011, Hardy and Tollefsbol coined the term “epigenetic diet” to refer to the dietary intake of all the compounds with protective properties against epigenetic modifications, including folates, isothiocyanates, isoflavones, resveratrol, curcumin, and tea polyphenols, among others (75). However, data on therapeutic options to reduce the impact of BPA are still quite scarce.

To the best of our knowledge, this is the first comprehensive narrative review on BPA-induced epigenetic changes and its consequence on male reproductive health. Indeed, we explored the effect of BPA in any aspect of reproductive system anomalies, considering different species. Furthermore, various epigenetic targets of BPA in reproductive disorders were also analyzed. On the other hand, we recognize that this led us also to a limitation, since we did not apply a systematic approach.

Given the relevant epigenetic effect of BPA and other EDCs, it could be useful in future to define specific epigenetic markers associated with male reproductive dysfunction during preconceptional analysis (8, 76, 77). In addition, since epigenetic changes can be potentially treated, target therapies could represent a very interesting topic of study in order to preserve fertility in subsequent generations.

## Conclusion

Exposure to BPA has the potential to induce epigenetic modifications in both animal and human cells. Such modifications could in turn play a role in male reproductive disorders and cancer development. An epigenetic transmission to offspring was also demonstrated.

Further research is needed to define the mechanisms underlying BPA-related epigenetic changes in paternal sperm and offspring phenotype and to find appropriate therapies to reduce the impact of BPA-induced dysfunctions.

## Author Contributions

FC, IS, and AC contributed to the conception and design of the study. AC, FB, and CC organized the database. FC and LC wrote the first draft of the manuscript. CB, IS, EA, SP, and CA wrote the sections of the manuscript. All authors contributed to the manuscript revision, read, and approved the submitted version.

## Conflict of Interest

The authors declare that the research was conducted in the absence of any commercial or financial relationships that could be construed as a potential conflict of interest.
